# Insights from an Intervention to Support Early Career Faculty with Extraprofessional Caregiving Responsibilities

**DOI:** 10.1089/whr.2021.0018

**Published:** 2021-08-23

**Authors:** Lauren A. Szczygiel, Rochelle D. Jones, Amelia F. Drake, Wonder P. Drake, Daniel E. Ford, Katherine E. Hartmann, Anne M. Libby, Bess A. Marshall, Judith G. Regensteiner, Kristine Yaffe, Reshma Jagsi

**Affiliations:** ^1^ Center for Bioethics and Social Sciences in Medicine, University of Michigan, Ann Arbor, Michigan, USA.; ^2^ Division of Pediatric Otolaryngology, Department of Otolaryngology/Head and Neck Surgery, University of North Carolina, Chapel Hill, North Carolina, USA.; ^3^ Department of Pathology, Microbiology and Immunology, Vanderbilt University School of Medicine, Nashville, Tennessee, USA.; ^4^ Institute for Clinical and Translational Research, Johns Hopkins School of Medicine, Baltimore, Maryland, USA.; ^5^ Department of Obstetrics and Gynecology and Medicine, Vanderbilt University School of Medicine, Nashville, Tennessee, USA.; ^6^ Department of Emergency Medicine, Center for Women's Health Research, University of Colorado School of Medicine, Anschutz Medical Campus, Aurora, Colorado, USA.; ^7^ Division of Endocrinology and Diabetes, Department of Pediatrics, Washington University in St. Louis, St. Louis, Missouri, USA.; ^8^ Department of Medicine, Center for Women's Health Research, University of Colorado School of Medicine, Anschutz Medical Campus, Aurora, Colorado, USA.; ^9^ Department of Psychiatry and Behavioral Sciences, Weill Institute for Neurosciences, University of California, San Francisco, San Francisco, California, USA.; ^10^ Department of Radiation Oncology, University of Michigan, Ann Arbor, Michigan, USA.

**Keywords:** caregiving, physician-scientist, academic medicine, intervention, career development

## Abstract

***Background:*** Insufficient support for balancing career and family responsibilities hinders retention of physician-scientists. Programs to improve retention of this important group of faculty are crucial. Understanding the experiences of program implementers is key to refining and improving program offerings.

***Methods:*** We conducted an interpretive, descriptive, and qualitative study as part of an ongoing evaluation of the Doris Duke Charitable Foundation's Fund to Retain Clinical Scientists (FRCS) awards. We conducted telephone interviews with 12 program directors representing all 10 US medical schools who received the Doris Duke funding in 2016.

***Results:*** Of the 12 participants, 10 were women (83.3%). Participating program directors perceived the FRCS award as capable of producing paradigmatic changes regarding how responsibilities at home and work in academic medicine are viewed and integrated by early-career faculty members. The main qualitative themes that captured directors' experiences implementing the program were as follows: (1) championing a new paradigm of support, (2) lessons learned while implementing the new paradigm, (3) results of the new paradigm, and (4) sustaining the paradigm.

***Conclusions:*** These findings may help to inform development of similar programs to retain and support the career progress of physician-scientists with extraprofessional caregiving responsibilities. The interviews illuminate ways in which the Doris Duke FRCS award has driven institutional culture change by normalizing discussion and prompted reassessment of extraprofessional challenges and how best to aid early-career faculty members in overcoming these challenges.

## Introduction

Physician-scientists play a key role in advancing scientific and clinical knowledge necessary to promote human health. A robust and diverse workforce of physician-scientists is necessary to optimally advance knowledge and bridge the gap between research and practice. However, retention of this valuable workforce has proved challenging.^1,2^ Data from the Association of American Medical Colleges (AAMC) indicated that the 10-year retention rate for first-time assistant professors was only 43%.^3^ Prior research suggests that inadequate support for managing career and family responsibilities may impair the retention of early career faculty and that this encumbrance is particularly relevant among women physician-scientists.^4–6^ Previous studies suggest disparities in extraprofessional responsibilities play an important role in driving differences in academic rank and productivity between male and female physician-scientists.^5,7–9^ Addressing physician-scientist attrition and gender disparities in promotion and retention in academic medicine requires the implementation and evaluation of creative interventions.^1,10^

To support faculty development and retention, in 2016 the Doris Duke Charitable Foundation (DDCF) launched the Fund to Retain Clinical Scientists (FRCS), an innovative, multisite program to support early-career physician-scientists facing extraprofessional caregiving challenges.^11^ Results from evaluation of this program have shown that recipients of the caregiver support awards perceived that the award meaningfully improved their productivity by ameliorating time challenges brought on by extraprofessional caregiving^12^ and lessened the stigma of caregiving challenges, particularly those associated with childcare and motherhood.^13^

Prior research about physician-scientist caregiver support awards has described faculty perceptions of the award on their well-being and on improving institutional culture,^12–14^ but little is known about the leaders' motivations and challenges experienced when implementing such awards. Therefore, we sought to explore leaders' perspectives in this study.

## Methods

### Context

The DDCF selected 10 US medical schools in 2016 to participate in its FRCS program, which aimed to enable these institutions to support early-career physician-scientists facing significant caregiving challenges. The selected sites then solicited applications from investigators at their institutions. Eligibility for individual awards required that faculty be MDs early in their careers (instructor or assistant professor), engaged in currently funded research with the potential to impact human health, and facing significant caregiving challenges. Individual faculty members who received the award from their participating institutions received funding of ∼$30,000–$40,000 to directly support their research. Although each site agreed to specific program elements, including the individual awards supported by DDCF funds only be used for research support (*i.e.*, funds could not be used to pay for childcare or eldercare), some sites introduced additional features using institutional or other philanthropic support that generated variation in implementation and experiences across sites. Full program details have been reported previously.^11–13^

### Study design

As part of an ongoing evaluation of the Doris Duke FRCS program, we conducted qualitative interviews with program directors. Our approach to this research was that of interpretive description.^15,16^ Specifically, we sought to identify patterns and themes in program directors' subjective experiences. Our goal was to describe and interpret the phenomenon of navigating the demands of implementing a unique and innovative grant funding mechanism geared specifically toward faculty with extraprofessional caregiving challenges.

We aimed to generate a description capable of informing the ongoing implementation of the FRCS award and future development of other interventions designed to improve work-life integration for physician-scientists. We invited all program directors and co-directors to participate in telephone interviews in late 2019/early 2020 and participation was requested from at least one leader from each site.

Site directors were deliberately included as both researchers and research participants in this study. By acting as “insider researchers,” site directors provided contextually embedded knowledge during the interview guide development, critical feedback on preliminary findings, and presentation of final results.^17^

Two interviewers (L.A.S. and R.D.J.), both trained in qualitative research methods, conducted the interviews. All participants were informed of the study procedures and granted verbal consent to proceed with the audio-recorded interviews. The University of Michigan Institutional Review Board determined this study to be exempt from review.

### Data analysis

We used thematic analysis to code the interview transcripts, identify patterns, and then organize the codes into themes and subthemes.^18,19^ Two analysts (L.A.S. and R.D.J.) analyzed the interview transcripts with all codes and themes reviewed and approved by the senior author (R.J.). We developed the initial codebook based on our previous knowledge of the literature, in combination with initial impressions from early interviews. We inductively generated additional codes through an iterative coding process. Each analyst independently coded all interview transcripts using Dedoose (version 8.3.17; SocioCultural Research Consultants, Los Angeles, CA) and then met regularly to compare and consolidate codes and resolve any discrepancies. We identified initial themes and subthemes by consolidating the codes according to patterns we observed in the data. Finally, we reviewed the initial themes in comparison with the entire dataset to ensure that they accurately reflected sentiments present in the dataset as a whole.^19^

In lieu of performing individual member checking (which has been discouraged as a method to improve rigor),^20^ we solicited feedback on the interview guide from site directors (including those who did not participate in interviews), and presented preliminary findings from the first 11 interviews during an annual meeting of the site leaders to engage in discussion of identified themes and support trustworthiness of the findings.

## Results

Of 17 site leaders invited, 12 representing all 10 US medical schools who received Doris Duke funding participated in interviews (70.5%). Ten of the participants were women (83.3%). Interviews lasted an average of 46 minutes (range 24–68 minutes).

We identified four major themes that described directors' experiences with implementing Doris Duke FRCS award program at their institutions: (1) championing a new paradigm of support, (2) lessons learned while implementing the new paradigm, (3) results of the new paradigm, and (4) sustaining the paradigm. We describe a selection of themes and subthemes in the results hereunder. Supporting quotations throughout the article are denoted by Q# and correspond to the full quotations provided in (Tables. 1–4).

**FIG. 1.**
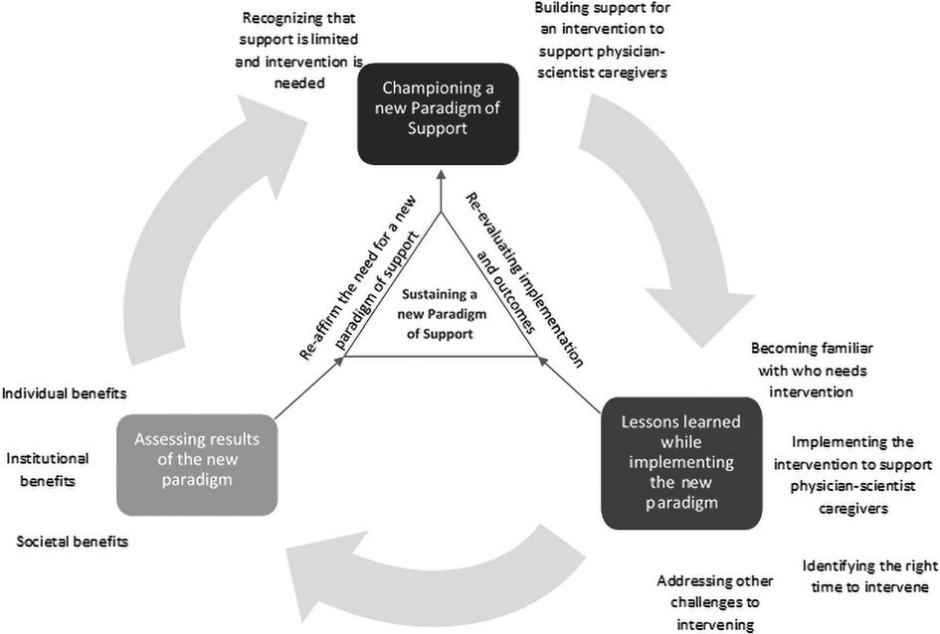
Thematic map of themes and subthemes. This thematic map illustrates the cycle that directors described of moving from program creation to implementation and then assessing the results of the program to drive further development of the intervention. It also illustrates the process of incorporating outcomes from results and lessons learned from implementation and directors' to plans use the gained information to sustain the interventions at their institutions.

The thematic map (Fig. 1) illustrates the cycle that directors described of moving from program creation to implementation and then assessing the results of the program to drive further development of the intervention. This thematic map also illustrates directors' descriptions of incorporating outcomes from results of the intervention as well as lessons learned from implementation and their plans use the gained information to sustain the interventions at their institutions.

**Table 1.** Theme 1 Supporting Quotations by Subtheme and Category

Theme 1: championing a new paradigm of support		
Subtheme	Category	Exemplar quotes
Recognizing that support is limited and intervention is needed		(Q1) *[M]edicine has this big mandate, you don't complain… [T]his program has really increased awareness that there are some life challenges that you can't just work your way through, and that they actually have the potential to derail your promising career…it's almost a normal thing now to realize that early career faculty have life/caregiving challenges…* (Female director) (Q2) *Prior to the existence of [the FRCS award] people with predictable episodic needs, … a very common example in my career mentoring was a young woman who was pregnant who had pregnancy complications and was put on bed rest for three or four months … And, all of a sudden, … the institution or the social group for that individual would have to either surround her and support her and make sure that her program projects moved forward while she was out or she just worked harder from home… So, I worked in an environment where we would under the radar, off the institutional radar,… support people like that. But we wouldn't speak about it publicly because it wasn't technically consistent with the policies of the institution. I think the existence of [the FRCS award] now makes this an officially sanctioned pathway for people like that who have an unexpected outcome, or even expected ones!* (Female director) (Q3) *The expectation that is built into academic medicine is that you pay to play. You … will forego your personal life … in order to be able to succeed in academic medicine…That is the paradigm that we have grown up with and so, this [FRCS] is a way of communicating it's not like that anymore… [A]cademic medicine is built on one salary supporting the labor of two people: one is the person with unlimited availability to work no matter what, and the other is the household engineer who manages everything and does the work of caregiving, social engagement, ethical community membership, remembering the social interactions that make a community a community. And that is the paradigm for success that we have built in academic medicine. The world isn't like that anymore. We don't have one salary supporting two people and we don't have household engineers – women certainly don't – some men do have those kind of marriages and it works really well. It really does support your ability to work, have unlimited availability to work if you have somebody at home, but that's more commonly available for men than it is for women.* (Female director) (Q4) *[The FRCS program] forces everyone to think about the issues about how you consider extraprofessional stress. What is the average amount of stress? What is exceptional stress? What is controllable stress? What is the difference between a short-term period of stress versus something that you can expect is going to go on for decades? So, I think that has been helpful in making the organization and the leaders think more deeply about the issues.* (Male director) (Q5) *I think there is greater recognition in the institution of extraprofessional demands. I think there is great appreciation of the grant and the [FRCS] program. I think that there is more awareness of what could be done to help support people…but I don't think we have done enough yet. I still feel like maybe we are 20% of the way there in terms of awareness and changing our policies, procedures, and our structure to make it better for people.* (Female director) (Q6) *I think another challenge has been that while [the Doris Duke program] opens up the discussion about work/life balance and caregiving challenges, there are many problems that are brought to the table that are not resolvable with this mechanism. As an example, …it would be really nice to have emergency or sick childcare…* (Female director)
Building support for an intervention to support physician-scientist caregivers	Gaining institutional buy-in to increase impact of the intervention	(Q7) *I hoped to…use [the FRCS] program to… encourage the faculty and department leaders and school of medicine leaders to begin to think more comprehensively in about what it means…to provide support for a faculty member throughout their career.* (Male director) (Q8) *I think that I would advise [someone implementing a program like the FRCS at their institution] to communicate with [their] chairs about it, to let them know what this award is all about and how it is different, how it is unique, the kind of person that it is geared toward to support. And I would encourage them to think about ways that they can provide matching support for the award recipients. I would talk to the dean about building in matching funds to begin to make it sustainable over time.* (Female director) (Q9) *I think we were really lucky because in a lot of cases where we would have had to discontinue funding [for an awardee just because the [FRCS] program didn't have enough money in it, in a lot of cases the [awardee's] department chair was aware of how … that funding had helped them and so the department continued the funding for an additional year … I think also another recognition that the program was of value.* (Female director)
	Building the knowledge-base to extend impact of the intervention to other institutions	(Q10) *The meetings [with other participating FRCS sites] have been very important because people have really interpreted this award in very different ways…. And so, it is helpful to hear how other people are viewing this and what their experiences are so, I have learned a lot from them about the culture, about their program but also the culture of the different institutions and how academic medicine is evolving based on experiences with this award …I mentioned that [a program leader at another participating FRCS site] thought about the applicants as a whole as all benefiting from support and I hadn't thought about that before…We adopted their approach to offering services to [non-funded] applicants as well.* (Female director)

FRCS, fund to retain clinical scientists.

**Table 2.** Theme 2 Supporting Quotations by Subtheme and Category

Theme 2: lessons learned while implementing the new paradigm		
Subtheme	Category	Exemplar quotes
Becoming familiar with those who need intervention		(Q11) *I think these extraprofessional demands are much more prevalent than anybody anticipated. We have heard incredibly compelling stories of devastating life issues, financial issues, mental health issues, physical health issues, big life stressors related to people's spouses, children, aging parents and also, things related to visas and the international and spread-out nature of people's families and caring for family members and responsibilities that I think are really impacting people's ability to keep their career going and stay in academics. And those disproportionately impact women and people of color. So, it's much worse than we thought. I mean I thought there was some of this out there, but I didn't realize there was so much!* (Female director) (Q12) *I think that the best stories, at least that hit both department chairmen and the school of medicine are a faculty member who has three children at home and is caring for parents. I would say this notion about the “sandwich generation” has come out a lot more in this protocol than people thought … and the amount of stress that occurs.* (Male director) (Q13) *[W]hat we have found is that people, even though they are applying for this, they really underestimate all the things that are causing them stress. They usually identify one thing but when you start asking them, you find out so many other things… They are applying because they had a child who was hospitalized for a month and now, has to go for therapy three times a week. But then you start asking and they [reveal]…my mother- in-law got sick and she came to live with us, and she is bedridden. … So, I think that we have … learned to try to … prompt the person a little bit so they will recognize all the things that they perhaps should be seeking help with. (*Female director) (Q14) *So, [prior to the Doris Duke program] both men and women who didn't perceive the institution as a place to seek substantive support for their careers … in these periods of [extraprofessional challenge],… just either fell back and they hoped that nobody noticed for six months to a year while they recovered. Or they …just changed their own visions for their careers. They reduced … their ambition to do science and tried to find something else.* (Female director) (Q15) …*[W]omen and people of color are very vulnerable to drop out and to leave and[they] face tremendous extra stressors in academic medicine… [Women and people of color] are the people who are most important to retain in science because they are already such a minority.* (Female director)
	Defining “need” and balancing scope of intervention	(Q16) *What becomes significant in our mind now is skewed toward the really devastating so if somebody has three kids under the age of three, or they have twins or they have triplets, and they have a working spouse and no family in town, that's actually incredibly stressful. But compared to brain cancer, it's like nothing. So, I think what has happened is that we are undervaluing the normal and significant stress of… having a career and having kids and that period in people's lives. So, I think we actually should be doing something for all those folks, but we are not. We are just saying, no, that's normal life; suck it up. You don't have brain cancer. When, in reality, you know, I think the distribution of these stressors really needs further analysis because how people cope with stress and their resiliency, their wellness, sleep deprivation, their physical, mental,… emotional health is impacted just by having kids, right? It doesn't have to be a disastrous pregnancy, it doesn't have to be a disastrous health issue for a child, but even just having small children is …a big life stress*. (Female director) (Q17) *My personal opinion is the institution, our academic medical center, should probably have in place…resources and support for employees and faculty that are easy to access. Things like relatively low-cost childcare, the ability to work from home,… the cultural acceptability of working from home at least some of the time, the ability to negotiate part time faculty positions that are still on the tenure track…Those were not culturally acceptable ideas. So, we were getting applications from people who would have benefitted from institutional resources, but our resources were really…designed for episodic needs. And we had a lot of those conversations about, you know, is anticipating a second baby with an otherwise healthy pregnancy enough of an extraordinary circumstance to warrant support? And I think most of us came down on the side of no, these are anticipatable personal challenges that everybody has but, boy,…it would be better if the institution had other resources in place.* (Female director)
Implementing the intervention to support physician-scientist caregivers		(Q18) *It's just how [FRCS awardees] feel about [the award], like my gosh! “This extra $40,000 just makes a huge difference. I can hire part of a research assistant or buy out this, and now, I don't need to be doing the original data collection on these subjects in my clinical trial or updating my biosketch or this other grant that I'm getting.” It adds the extra helping hands that they need and then, provides them with the time…So, you use the money to help give yourself time so that your time is blocked out to do high-level stuff, right?* (Female director) (Q19) *[W]e created some travel grants so that if they don't have money to present their research somewhere, I think we put together either two or three of these each year, of about [$]1,500 a year…. they just present a reason that they would like to use a travel grant to support their research, so that …they can network about future opportunities and all the stuff that goes with that type of thing.* (Female director) (Q20) *I think we also are very supportive of our scholars. We don't just give them the money. We provide mentoring opportunities for them. We provide trainings for them. We are involved with them. We don't just hand them money and say, good luck.* (Female director) (Q21) *Regarding what you are asking about how Doris Duke specifically, how that grant has helped…. what it has created is a cohort. So, we just had our first call with the next who are joining this group and they say really what they want is they want to be together, you know. It can feel isolating and when you have something like a really sick partner or parent or child that you are caring for at home it can feel like you are the only one that's going through this. … And so, coming together and learning … life hacks together is something we have been talking a lot about, and using their experiences to help others. You know altruism is a strong driver and a very good healthy defense for when things are rough in life and how they can work together to advocate for things to make it easier for other caregivers.* (Female director)
Identifying the right time to intervene		(Q22) *I think the other thing that makes a sweet spot is that the help they need is usually …[a part time] person [to assist with their work] and if they have access to part of a person right away that they can hire this month, you know, then it works It's hard to assess that in the application process but we are just realizing how … people have been so successful and so have been able to jump over to their RO1 funding, you know, who were they, how did they use the money; how quickly did they use the money.* (Female director) (Q23) *And to contrast, it sounds kind of obvious, but to contrast this or to give you a sense of contrast, you know, we have given money to somebody who had a series of unexpected crushing life events and there continued to be additional life events after the award was made. …[We] are struggling with him getting around to being able to spend the money because he is so consumed by… these crises… And so, the sweet spot would have been if he was all ready to go… but he wasn't quite at that point.* (Female director)
Addressing other challenges to intervening		(Q24) *I think one of the things that we have struggled with here is the tension between giving people support so they can buy out [clinical] time, where they can have additional support to make up for the time that they need to be with… an ill family member or sick family member or family member who is in another country, and us asking too much of them in return. So, we have tried to just thread that needle like,…we would like to offer people a whole panoply of … services but what we have to be able to do is be respectful of their time, because that's really what they need, they need time.* (Female director) (Q25) *I think a challenge has been… knowing how much to try to get involved in the life and work [of] the award recipients, and to try to help them in whatever way we can … versus just giving them the money. And I think we want to be there for them, we want to do what we can…they have good mentoring already and so, we get the message that they don't want additional obligations because, as a matter of fact, the problem they are having is work/life balance challenges, time management challenges.* (Female director) (Q26) *I think that another surprising [observation] to me is that it seems to me that faculty when they tell the story of the extraprofessional stress … [They] have just been less willing to talk about how the stress has impacted on them personally. And I think it just goes into what's a safe story to tell at work and what is a less safe story to tell at work…what the safe story to say is I am a very effective faculty member. I am working really hard… It's a lot less safe to say, again, that “this has been going on for years. It has demoralized me. I am really depressed. I'm getting treatment for my depression, and I now realize I have to reorganize the way I work to be more efficient.” That is the unsafe story because it kind of implies you are not a reliable partner or you have some inability to cope which could be a problem… And I'm sure there are some people doing the calculation that would say, “I don't think it's worth the $20,000 a year if I have to reveal all that to my colleagues and department chairs.” I have heard that kind of, you know, informally.* (Male director) (Q27) *I think the sort of challenge we have is that for every one person we have helped, I think there are probably five or 10 more people out there who have equal situations that need help that are not applying because they still don't think that their need is as great as others or they don't feel comfortable disclosing what their personal or family health challenge is.… [D]octors are kind of trained to ‘suck it up’ and be resilient and never complain and just power through it. So I think that they still don't think they need help or they think asking for help is a sign of weakness or an admission that they are not able to make it when, in reality, they are doing this heroic thing already.* (Female director)

**Table 3.** Theme 3 Supporting Quotations by Subtheme

Theme 3: assessing the results of the new paradigm	
Subtheme	Exemplar quotes
Individual benefits	(Q28) *[The FRCS award recipients] go from being potentials to being amazingly successful…. The term that I thought of just while we were talking was “rocket fuel” just rocket fuel for these people. And could they have done this anyway? I would say no, because what happened is that their data acquisition rapidly accelerated, their data analysis rapidly accelerated, their grant submissions happened when they would not have happened otherwise, happened much sooner because of the award. And so, it accelerated everything that you need for success.* (Female director) (Q29) *I think [FRCS awardees] are publishing a lot more…I think they are able to maintain their career trajectory. They gain additional sources of funding… I have seen that it has increased their confidence that they would succeed in academic publishing which is just really encouraging.* (Female director) (Q30) *Some of [the FRCS program participants] have achieved the independent funding that they need to make their career really secure and progress. Many of them have used their funding to hire help with their research, either a lab technician or a study coordinator …which has been helpful for them in accelerating the rate of their research so that they have been able to complete publications and move themselves forward to meet goals that they need to remain physician-scientists and not either have to leave the university for another institution or change their career path from physician-scientist to something else. So, I think we have seen good success.* (Female director)
Institutional benefits	(Q31) *And then, there's a return on investment so we put a really small investment in and got a huge investment out and so we got a great ROI so, affecting the bottom line has been huge, right?…That has been very important consequence that I think could be leveraged even more.* (Female director) (Q32) *… I think that division directors,… if one of their members applied and for some reason didn't receive the [Doris Duke FRCS] support, would have figured out some way to do it within their internal budgets because now, they have an institutionally sanctioned model for doing that. It was like it just gave us that wedge, you know, before and after. And after there is now a model and this is how we behave…And so, I think we are in a different era now and I think that's hard to tell how much of that is, you know, general trends but I do think the existence of the [Doris Duke FRCS] fund was critical… It looks like another one of those grant applications that is available to people. It's in the model of an academic institution so that's good. It's not just a HR or employee health program but it's managed by scientists for scientists, and it sets the culture in a different direction.* (Female director) (Q33) *[The Doris Duke FRCS] has had… a subtle effect of …normalizing the challenges that otherwise, people had tried to keep out of the work place; challenges that they were having at home. I think there was a sense that should be separate. You shouldn't bring those to work, that you should be strong enough to overcome those things and not bring them to work with you. And I think that the Doris Duke award helped us to further demonstrate institutionally that we believe in this but also, it's like a validation when the external funding agency says this is important enough that we are going to put funding up for this. And so, it's a form of external validation for everybody to see that there has been an important funding agency putting a stake, a flag in the ground about this.* (Female director) (Q34) *I think the institution as a whole is realizing that there should not be any stigma associated with facing a life caregiving challenge… we have tried really hard to let [Doris Duke FRCS program participants] know that this happens to everybody and in the past we just tried to work our way through it. … So, I think they feel very supported which boosts their resiliency. I think it's almost becoming a cultural norm…I think it's like a cultural shift.* (Female director)
Societal benefits	(Q35) *I think these scholars' families benefit tremendously, not only the people they are giving care to but their spouses.* (Male director) (Q36) *Where both parents are working, you know, somebody has to take the time [to take child to medical appointments]. So, very time consuming, it's not just one visit; it's multiple. So, this [child] whose mother is able to go with her to those visits, hugely benefits … it's a big life deal and instead of being negative, the little girl is becoming an independent strong thinker herself…*(Female director) (Q37) *I think the scientific community is benefitting because the faculty are maintaining their productivity throughout the caregiving challenge …* (Female director)

**Table 4.** Theme 4 Supporting Quotations by Subtheme

Theme 4: sustaining a new paradigm of support for physician-scientist caregivers	
Subtheme	Exemplar quotes
Reevaluating implementation and outcomes of the intervention	(Q37) *I think it's really important to get the highest level of engagement of the key leadership, … on the research side, on the dean's side, on the physician-scientist training program side so that you have lots of points of contact. And then also the points of contact with the people who do faculty development and CTSA so that you are not recreating things that already exist, or that you are not building another silo. So, I think it's really important to figure out how to integrate this fully into the architecture of the school …and then to find all the ways to connect with departments and multiple microenvironments, and then ways to support and enable people without making extra work for them.* (Female director) (Q38) *So, the one part that we are … starting to look at is our mentoring set up, whether that's being successful. So, we are surveying our applicants and awardees right now to… get an idea how frequently they are meeting with their mentors, how successful that has been, … what they might find more, or less helpful. …I think a lot of our decision-making will be based on looking at the success of our awardees and … other institutions' awardees, and trying to tweak our program to improve what we have got.* (Female director) (Q39) *I think it probably also benefitted the institution in … shining a light on this problem. So, the Doris Duke Foundation, of course, doesn't fund anything other than human research so I think the success of this program, … has raised the institution's interest in starting a program of its own for people who are doing non-human research and people who are basic scientists rather than physician-scientists … Hopefully, the institution will start an expansion based on this program, an expansion that would be available to people that wouldn't be eligible for the Doris Duke funding.* (Female director)
Reaffirming the need for a new paradigm to support physician-scientist caregivers	(Q40) *You know once we have a little bit more data … we need to show those outcomes. I will present that to our dean and our research council and say, look, we believe we have the data to show this is a successful program and I think we will either look potentially for philanthropic [methods of funding the program] and … work with departments, particularly large departments, that… could do a similar program within their own department.* (Male director) (Q41) *I would say that meeting with … whoever at your institution would be responsible for taking in extra funding – to meet with them and nail down what they would be willing to do. Would they be willing to match funds? Would they be willing to extend at the end? Would they be willing to pay for programs; that kind of thing because I think that has been a major part of our success, is that we have been able to sort of double our funding or even quadruple our funding in some cases.* (Female director)

CTSA, Clinical and Translational Science Award.

### Theme 1: championing a new paradigm of support

#### Recognizing that support is limited and intervention is needed

Directors acknowledged a preexisting paradigm within academic medicine that prioritized physician-scientists sacrificing their personal lives in favor of productivity and stigmatized seeking support for extraprofessional challenges (Q1, Q2).

One director described this paradigm as outdated and built on the premise of the labor of a two-person household; the male physician-scientist who was expected to prioritize research productivity and patient care and the physician's partner (typically a woman who may not work outside the home) who supports the physician-scientist's ability to work by managing caregiving and domestic tasks (Q3). This outdated paradigm was perceived as unsustainable, given the current high numbers of physicians who do not have a supporting spouse upon whom they can rely.

Some directors perceived that lack of resources to address or acknowledgement of extraprofessional challenges from their institutions stigmatized discussing work-life conflicts and that the Doris Duke award ameliorated some of this stigma. One director stated that the perceived stigma forced faculty members to seek support “off the institutional radar” (Q2) but that the Doris Duke award offered an “officially sanctioned” way to encourage early-career faculty members to open up about their extraprofessional challenges and seek support (Q2). The Doris Duke award explicitly promoted a paradigm of support by encouraging institutional leaders to discuss caregiving and the way it affects faculty members (Q4).

Although praise for the Doris Duke program was unanimous, some directors still articulated a need for more progress in furthering a supportive paradigm within academic medicine and for institutionally embedded support for physician-scientist caregivers, such as greater availability of parental leave and emergency childcare leave (Q5, Q6).

#### Building support for an intervention to support physician-scientist caregivers

##### Gaining institutional buy-in to increase impact of the intervention

Bolstered by the successes achieved in the early stages of the program, many directors recognized the need to build institutional support to broaden the reach and impact of the program through the assistance of institutional leaders (Q7). Soliciting matching funds from awardees' departments or through other funding mechanisms at the institutions was a common way of obtaining additional support (Q8, Q9). Directors who successfully solicited additional support credited the demonstrable successes of scholars and “a recognition that the program was of value” (Q9). This approach also sent the message that the institution was willing to participate in solutions and share responsibility for caregiving demands.

##### Building the knowledge base to extend impact of the intervention to other institutions

Program directors described the importance of demonstrating positive outcomes and impacts of the award and sharing lessons learned at their site with other FRCS directors. Capitalizing on program successes at each individual institution, directors shared their strategies with each other at the FRCS directors' annual meetings. These meetings enabled directors to collaborate with each other and expand knowledge of the needs and priorities of early-career physician-scientists (Q10).

Directors' descriptions of the importance of support from institutional leaders and other site directors demonstrated a recognition that while the Doris Duke program enabled them to implement an intervention to support physician-scientist caregivers, broader institutional support and demonstration of positive outcomes such as increased extramural funding, increased research productivity, and improved well-being were necessary in promoting a new sustainable paradigm for physician-scientist caregivers.

### Theme 2: lessons learned while implementing the new paradigm

#### Becoming familiar with those who need intervention

Implementing the Doris Duke program allowed directors to deepen their familiarity with the needs of their assistant professor faculty. Directors perceived a strong need for support for work-life integration among early-career faculty members at their institutions that was “more prevalent than anybody anticipated” (Q11). Many FRCS applicants experienced multiple caregiving challenges at the same time.

For example, in addition to childcare, many faculty members were also responsible for their aging parents, managing domestic responsibilities, and taking care of personal health care needs (Q12). One director described that even when applying for the award, many early-career faculty members either understated or underestimated the number and impact of stressors in their lives (Q13).

Another reason why faculty needs may have gone unnoticed before the Doris Duke program was the perceived stigma in disclosing extraprofessional challenges. One director described faculty members who “flew under the radar” because of the stigma associated with revealing caregiving challenges and instead of seeking support, lowered their career ambitions or left the research track entirely (Q14). Women and underrepresented minorities were perceived as particularly vulnerable to the compounding challenges of balancing work and extraprofessional challenges and therefore more susceptible to burnout and attrition (Q15).

##### Defining “need” and balancing scope of intervention

Given that the Doris Duke funds awarded to each institution were intended to support two to three award recipients each year at each site, directors and award committees faced difficult assessments of which applicants' needs were more significant when making award determinations. One director described how her views of what constituted significant need began to “[skew] toward the really devastating” (Q16). This skew led to faculty members with more typical caregiving challenges, such as those associated with child-rearing, to being judged as less critical than those with challenges such as terminal illnesses in the family or other similarly disruptive challenges (Q16).

Lack of institutional support and policies to support personal flexibility for faculty members with typical parenting challenges and the difficulty of supporting this population through the Doris Duke program was perceived as a key limiter of the scope of the intervention (Q17).

#### Implementing the intervention to support physician-scientist caregivers

Once it was determined which faculty would receive the FRCS award, the primary method of intervention was direct funding for research needs (funds could not be used to pay for caregiving expenses). These funds were intended to free up valuable time for scholars by providing them the means to hire support staff, or have salary support or clinical buyout that enabled them to spend more time engaged in their research endeavors (Q18).

In addition to direct funding, many sites developed novel complementary programs and services for FRCS scholars. These auxiliary services, provided using institutional funds, varied between sites and included small travel grants, support for grant writing and manuscript editing, and small grants for childcare services (Q19, Q20). Some sites even made these supplementary resources available for faculty who had applied for but not received the FRCS award or who were not eligible for the award, thus extending the reach of the support paradigm to a broader range of faculty (Q21).

#### Identifying the right time to intervene

Directors discussed the importance of timing when making award determinations. The idea of timing referred to identifying potential award recipients who were at the ideal time in their careers and in their research projects to make the most efficient use of the FRCS funds.

One director described the ideal timing as an applicant being at a point in their research where they could quickly put the award funds to use: (1) they had most of their research operations already established and (2) mostly needed additional personnel to aid with more tedious tasks such as data collection or data entry and they had a well-defined research plan (Q22). This director contrasted a “sweet spot” applicant with applicants who were not perceived as ideal award recipients owing to not being able to make use of funds because of considerable extraprofessional caregiving challenges (Q23).

#### Addressing other challenges to intervening

Challenges that directors faced in implementing the intervention included finding the right balance of additional resources and enrichment experiences without requiring too much time from awardees (Q24, Q25). Some directors also voiced concerns that young faculty members may not be seeking support because of the perceived stigma of disclosing their extraprofessional challenges and asking for assistance (Q26, Q27).

### Theme 3: assessing the results of the new paradigm

The FRCS program was perceived as having far-reaching effects for awardees, their families, and their institutions.

#### Individual benefits

Directors noted an extraordinary level of program impact for awardees, including improving awardees' ability to perform research data collection, article publication, grant attainment, and scholar-reported improvements in work-life integration and psychosocial wellbeing (Q28–Q30).

#### Institutional benefits

Some directors noted the possibility that the FRCS award had a positive impact on their institutions more broadly. One director suggested that the award had a significant return on investment, implying that the $30,000–$40,000 in grants to individual FRCS awardees could result in significantly more external grant funding for the scholars and the institution in the long run (Q31).

Of note, some directors commented on how the FRCS award improved the institutional culture by creating an “institutionally sanctioned” model for supporting faculty experiencing extraprofessional demands and other life stressors, with the potential to inspire other departmental support for faculty by acting as a model program (Q2, Q32). A few directors also noted a reduction in the stigma of discussing work-life integration issues as a benefit that signified a paradigm shift occurring in academic medicine (Q33, Q34).

#### Societal benefits

In addition to the direct benefits to scholars and their institutions, directors noted benefits the Doris Duke award had for scholars' family members and the broader community. One director noted that scholars' family members and others who were dependent on them benefitted from the increased amount of time that the scholar had to spend on caregiving and domestic tasks. This included scholars' family members (children, partners, and parents) who benefitted from the increased amount of time that scholar had to spend in their home lives (Q35, Q36). One director also noted that the broader scientific community of society benefitted from the awardees being retained as physician-scientists (Q37).

### Theme 4: sustaining a new paradigm of support for physician-scientist caregivers

Directors' comments indicated that shifting the paradigm toward support of work-life integration for physician-scientists was an ongoing process. The thematic map (Fig. 1) illustrates the roles that sustaining the new paradigm of support through reevaluating and improving program offerings and reaffirming the value of the intervention to institutional leaders played in further championing a paradigm of support. Reevaluating and reaffirming the paradigm of support were key in identifying new opportunities to continue the cycle of intervention creation, refinement, and sustainment.

#### Reevaluating implementation and outcomes of the intervention

Directors explained that frequent evaluation of program effects was key in identifying new ways and new groups of faculty to promote the program, offer new resources, or reassess award criteria. These lessons learned during program implementation (as discussed in Theme 2) strengthened directors' ability to champion a new paradigm by recognizing new opportunities and interventions to support physician-scientists with caregiving needs. Some directors described soliciting feedback from FRCS applicants and awardees as essential to improving program efficacy (Q5). For example, directors sought ways to make more efficient use of the FRCS funds by identifying resources already provided by their institutions such as faculty development, coaching or administrative support, and connecting applicants to those (Q38, Q39).

In addition, the FRCS award was only available for early faculty members involved in human research; however, some sites received interest from later career faculty members or faculty involved in nonhuman research who were also experiencing caregiving challenges but were not able to be served by the FRCS funding mechanism (Q40). Site directors discussed plans to extend the benefits of the new paradigm by identifying more efficient ways to intervene through incorporating the award into existing institutional supports and identifying those who were not having their needs met through the Doris Duke funding.

#### Reaffirming the need for a new paradigm to support physician-scientist caregivers

Directors worked to sustain the new paradigm of support by reaffirming the necessity of a new approach to supporting physician-scientist caregivers. Directors described plans to present outcome data to institutional leaders to advocate for the success of the program and seek other philanthropic or institutional funds or services to sustain and expand the program (Q41, Q42). Reaffirming the importance of the Doris Duke FRCS intervention to institutional leaders was described as an essential means of continuing to champion the new paradigm (as discussed in Theme 1) and creating additional ways to increase program reach and impact.

## Discussion

In this qualitative interview study, we examined directors' perceptions of a novel national program intended to support early career physician-scientists experiencing extraprofessional caregiving responsibilities and their experiences with implementing the program at their institutions. Our results show that site leaders perceive the program can produce changes regarding how home and work life in academic medicine are viewed and integrated by early-career faculty.

Some directors recognized that new cohorts of physician-scientists desire a culture in academic medicine that is more tolerant of the need for flexibility in managing life and work demands. They noted that an outdated paradigm within academic medicine supported a work culture that relied on the domestic work of one spouse, typically a woman who was not employed outside the home, to support the research productivity of the physician-scientist, typically a man.

With increasing representation of women in the physician-scientist work force,^21^ this paradigm of relying on a supportive spouse is increasingly less feasible for younger generations of physician-scientists. First, women are less likely to have stay-at-home spouses compared with men. Moreover, even in partnerships where both spouses work full-time out of the house, earlier research has shown that women still spend more time on domestic tasks and childcare as compared with their male counterparts.^4,5^

Second, the caregiving demand placed on physician-scientists who belong to “Generation X” or older Millennials has increased because of the necessity of caring for aging parents in conjunction with childcare and other domestic tasks. Studies have shown that caregivers within this dual role, also called the “sandwich generation,”^22^ experience higher levels of burnout and depression, particularly women.^23,24^ Several of our respondents indicated that the paradigm of relying on a spouse for support is unsustainable in academia and more institutional support for time flexibility and family leave owing to caregiving is needed to retain physician-scientists. Research on “sandwiched” caregivers within academic medicine is needed to understand the lived experiences of these faculty members, how they manage dual caregiving roles, and what steps can be taken to support them.

Several directors believed that situating the Doris Duke award within existing institutionally supported funding mechanisms, such as Clinical and Translational Science Award units, or gaining contributing support from institutional leaders, was an important factor associated with reducing stigma associated with discussing caregiving challenges. Prior research has documented “bias avoidance,”^25^ where individuals attempt to avoid career penalties by minimizing or hiding caregiving responsibilities because of the perceived stigma of being seen as not completely devoted to work.^26^ Prior research has shown that recipients of caregiver awards perceive these awards as indicative of a culture shift toward validating caregiver needs.^13,14,27^

The current results further support the idea that the benefit of the Doris Duke award lies partly in the fact that it is awarded for research merit as well as caregiving need. This dual purpose works to reduce stigma and augment the normalizing of the paradigm shift toward supporting work-life integration and away from expecting all support to derive from within the faculty member's family unit.

In addition, research in the program evaluation field has identified “institutionalization,” the routine integration of programs into existing organizational structure, as an essential component of sustainability.^28^ This would indicate that integrating the FRCS intervention with established institutional processes would serve to strengthen the likelihood of a paradigm shift toward support being maintained.

This institutionalization was also an important method of sustaining the paradigm by creating additional avenues for funding the program. Directors explained that soliciting additional funds from their institutions was an important method of ensuring program sustainability, considering that they were entering their last year of funding from the Doris Duke Foundation. Several directors had already put this plan into action by integrating institutional funds with Doris Duke Foundation funds through matching funds to support additional scholars and supplementary programs. Directors also discussed plans for soliciting further funds from other philanthropic organizations.

These findings may help to inform development of similar programs to support retention and productivity of physician-scientist caregivers by illuminating ways in which the Doris Duke FRCS may have driven institutional culture shift. Furthermore, programs aimed toward support and retention of women faculty members are particularly important in light of the COVID-19 epidemic. School closings, shelter-in-place orders, and social distancing have made caregiving and childcare more difficult to manage, exacerbating challenges already faced by early-career faculty members, particularly women. By limiting in-person connections, these measures have also decreased collaboration, networking, and mentorship opportunities that are critically important for early-career faculty.^29^ A recent National Academies report has synthesized evidence raising concerns that the COVID-19 pandemic will lead to a quantifiable decrease in the productivity of women faculty,^30^ including both commentary^31^ and bibliometric analyses.^32^ Institutions need to take deliberate action to support women to ensure that the existing gender gap in academic medicine does not continue to widen.^33^

Programs such as the Doris Duke FRCS that affirmatively address caregivers' challenges will be essential to supporting the work of women in academia. Indeed, the Doris Duke Foundation recently announced plans to offer additional grants through a special “COVID-19 Fund to Retain Clinical Scientists” program.^34^

Strengths of this study include a diversity of settings represented by site directors in terms of institutional size and geographic location.^11^ This research also benefits from careful use of qualitative data collection and analytical methods.^15–17,35,36^

We obtained a rich, descriptive dataset, and we reached thematic saturation, the point at which no new codes were identified with subsequent interviews, with the sample providing enough data to support the emergent themes.^37^ Data saturation, the point at which we had captured the participant's full understanding of the phenomena being studied, was achieved and aided by the directors' role as participant researchers. Directors' intimate knowledge of the program helped to narrow the focus of the interview guide and enabled the interviewers to fully capture program details and participant experiences. In addition, directors' feedback during the annual meeting aided in achieving saturation by confirming that themes that were identified resonated with their experiences.^37^

Limitations include that the work is qualitative and situated in a small number of institutions. Nevertheless, although some of our findings are specific to the population studied, we believe our study has broader policy implications for institutions interested in implementing programs similar in nature to the Doris Duke FRCS award. Finally, although some may argue that insider researchers may introduce bias because they are too close to the subject at hand, in the context of this study, engaging site directors as researchers and participants led to robust findings owing to directors' embedded knowledge and understanding of the Doris Duke program at their institutions.^17^

## Conclusion

Directors responsible for implementing the Doris Duke FRCS award at their respective institutions described perceptions that the award is effective in alleviating burdens associated with extraprofessional caregiving challenges for early-career faculty members. In addition, they believed that the award was capable of creating a paradigm shift in the culture of academic medicine that validated the needs of physicians with caregiving challenges and supported a healthier approach to work life.

The COVID-19 pandemic and its associated disruptions have recently heightened awareness of work-life conflict and its disproportionate impact on women in medicine. Although this study and the program it evaluates pre-date the pandemic, the lessons it illuminates are particularly timely as many institutions seek models for how best to support faculty to integrate their professional and extraprofessional caregiving demands.

## Abbreviations Used

AAMC: American Medical Colleges
CTSA: Clinical and Translational Science Award
DDCF: Doris Duke Charitable Foundation
FRCS: Fund to Retain Clinical Scientists
